# Determinants of new drugs prescription in the Swiss healthcare market

**DOI:** 10.1186/s12913-017-2775-1

**Published:** 2018-01-09

**Authors:** Anne Decollogny, Romain Piaget-Rossel, Patrick Taffé, Yves Eggli

**Affiliations:** Institute of Social and Preventive Medicine, Centre Hospitalier Universitaire Vaudois and Faculty of Biology and Medicine, Route de la Corniche 10, 1010 Lausanne, Switzerland

**Keywords:** Drug market, Drugs prescription, Prescription drivers, Non-hierarchical multilevel model, Bayesian Markov Chain Monte Carlo methods

## Abstract

**Background:**

Drug markets are very complex and, while many new drugs are registered each year, little is known about what drives the prescription of these new drugs. This study attempts to lift the veil from this important subject by analyzing simultaneously the impact of several variables on the prescription of novelty.

**Methods:**

Data provided by four Swiss sickness funds were analyzed. These data included information about more than 470,000 insured, notably their drug intake. Outcome variable that captured novelty was the age of the drug prescribed. The overall variance in novelty was partitioned across five levels (substitutable drug market, patient, physician, region, and prescription) and the influence of several variables measured at each of these levels was assessed using a non-hierarchical multilevel model estimated by Bayesian Markov Chain Monte Carlo methods.

**Results:**

More than 92% of the variation in novelty was explained at the substitutable drug market-level and at the prescription-level. Newer drugs were prescribed in markets that were costlier, less concentrated, included more insured, provided more drugs and included more active substances. Over-the-counter drugs were on average 12.5 years older while generic drugs were more than 15 years older than non-generics. Regional disparities in terms of age of prescribed drugs could reach 2.8 years.

**Conclusions:**

Regulation of the demand has low impact, with little variation explained at the patient-level and physician-level. In contrary, the market structure (e.g. end of patent with generic apparition, concurrence among producers) had a strong contribution to the variation of drugs ages.

## Background

New medicines have been associated with increased longevity on an international scale and, thus, recognized as a possible vehicle for both medical progress and quality of life improvement for patients [1]. In Switzerland, a recent study analyzed the impact of cardiovascular drug innovation on the longevity of elderly people and it was found that those “who used newer cardiovascular drugs in 2003 had longer time till death”, controlling for several demographic and health status characteristics [2].

Other authors stated that the substantial number of innovative pharmaceuticals introduced to the Swiss market should be examined in regard to the higher costs they generated [3]. Moreover, significant variations among cantons’ healthcare costs have been observed [4], which raises the question of fair access to new products. Thus, researchers and policy makers are interested in finding ways to control factors that influence physician’s adoption of new medications (see, e.g., this paper on mental disorders drugs [5]).

One additional motivation to focus researches on the determinants of new drugs prescription is that innovation and successful diffusion of new drugs are critical for financial performance of pharmaceutical companies [6]. In 2005 alone, 623 new drugs were registered on the Swiss Specialities List (a list published by the Federal Office of Public Health, which contains all the drugs reimbursed by the basic health insurance [7]), generating a significant review and administrative work.

For all the aforementioned reasons, we found it important to deepen the current state of knowledge about the mechanism yielding to new drugs prescription, by studying what are the main drivers of this novelty in the Swiss healthcare market.

Drug innovation can take different aspects [8]. A first distinction should be made between an innovation implying a new substance and one that is a reformulation of an already existing drug.

In the first category of innovation (i.e. new substance), one further distinguishes between a structural innovation**,** which denotes a drug with a new chemical structure of the active substance and a pharmacological innovation, defining a drug that either focuses on a new therapeutic target, provides a new mechanism of action in the body to treat the disease, or causes fewer or different undesirable side effects. This first category of innovations can either represent clear innovations (first-in-class drugs) or follow-on drugs (subsequent class entrants) [9], often considered as possible, yet imperfect, substitutes [10].

As for the second category of innovations (i.e. reformulation), pharmaceutical innovation brings new properties to a known and already marketed drug, while pharmacokinetic innovation offers a new and more appropriate profile for the absorption or elimination of this product [11]. Novelty in terms of formulation can be related to the galenic form (pill, coated table, granules, powder, etc.), the route of administration (oral, topical, sublingual, inhalation, injection, etc.), the dosage, or the number of doses. The purposes of such reformulation or line extension drugs can be manifold: facilitate administration and the comfort of the patient, adapt the dosage and facilitate observance of the treatment, and, possibly, diminish the impact of market loss at patent expiration by preventing competition from generics [12].

Note that new drugs might also enter the market although bringing neither a new substance, nor a new formulation. This is the case of a generic drug proposed by a new manufacturer, in a situation of co-marketing, or under a new brand.

Semantically, one talks about clinical innovation when this latter brings an added benefit over previous treatments, reduces side effects and, thus, leads to better therapeutic benefit, or when it is more adequate for certain patients’ profiles. However, the frontier between “true” innovation and comparable new drug is tenuous and incremental innovation, even modest successive modifications, may finally be considered as innovative [13].

This difficulty of defining true innovation is reflected in the complexity of disentangling innovative drugs among the newly registered medicines. Part of the controversy may arise from how innovation should be assessed [14] (i.e. looking at the drug’s therapeutic value, its economics aspects, its patents development, or simply regarding new drug counts [15]). Given the complexity of drugs innovation and the absence of consensus as regards ways to assess it, we tried to facilitate things by capturing the novelty of a pharmaceutical product using its registration date in the Swiss Specialities List (i.e. we considered that a drug was new if it was newly registered on the Swiss Specialities List, without distinction of any kind).

Regarding the drivers of this innovation, we differentiated between five types of determinants: those related to physicians, patients, substitutable drug markets, drugs, and regions (i.e. Swiss cantons), according to previous knowledge on this subject [6]. For each of these five categories, several variables were measured.

Physicians were analyzed regarding their clinical area (specialist or generalists) and their affiliation (hospital or installed practice). Patients were characterized by their age, gender, insurance deductible (which might influence the amount of patients’ co-payment) and a multi-morbidity index.

Substitutable drug markets are markets in which drugs are substitutable between each other. Even if there are some exceptions, for instance to avoid some drugs interactions or specific side effects for some patients, we considered all drugs having the same 4th level of Anatomical Therapeutic Chemical (ATC) [16] code to be substitutable and, thus, the substitutable markets were simply markets containing drugs with the same ATC 4th level code. Possible drivers analyzed for the markets were related to their structure (i.e. competition aspects [17]), their size [18] (in terms of treated patients, prescriptions and active structures available), as well as the expensiveness of their treatments.

Regarding drugs, we differentiated between over-the-counter and prescription drugs (Rx) and between generic and non-generic drugs [19]. We also added cantons dummies to capture cultural or marketing variables, which might influence regional prescription habits in different cantons, although drugs legislation is defined at the Swiss federal level [20].

Our analyses were based on Swiss health insurances data, which included over 2.8 million prescriptions. In a first time, we partitioned the total variation of the novelty prescription into five variance components: drug, substitutable market, patient, physician, and region. This allowed us to identify the largest source of variation and, therefore, of novelty. Then, we sharpened our analysis by assessing the amount of variation explained by the measured drug’s, market’s, patient’s, and physician’s characteristics, as well as by the cantons’ dummies.

## Methods

### Studied population

Our source population came from four sickness funds and consisted in 473,886 insured living in Switzerland in 2006. Since only drugs delivered by pharmacists are systematically recorded in Switzerland, the studied population was restricted to cantons prohibiting doctors’ drugs delivery (Aarau, Basel-Stadt, Fribourg, Geneva, Jura, Neuchâtel, Ticino, Valais and Vaud). For each drug, we had knowledge about its active ingredient (medicinal product), as well as the information available on its marketed package (i.e. its brand name, producer, formulation, pharmaceutical form, dosage, and quantity of doses). All analyzed drugs were listed on the Swiss Specialties List.

Each observation in our dataset represented a separated drug prescription, made by a specific physician, for a given patient (Table 1). If a physician prescribed the same drug for the same patient, it was considered as only one observation. A drug with the same active substance but with a different dosage, formulation, or quantity of doses in the package represented a new observation. If a different physician prescribed the same drug, we considered it as a separate decision and treated it as a new observation. Our goal was to capture the intention of prescription.

**Table 1:** Example of observations of different prescriptions

Patient	Number of boxes prescribed	Drug	Active ingredient	Market	Physician	Canton
Patient A	1	Brand X 10 tablets 500 mg	N02BA01	N02BA	Dr MD 001	Aarau
Patient A	3	Brand X 10 tablets 500 mg	N02BA01	N02BA	Dr MD 002	Bern
Patient A	1	Brand X 20 tablets 500 mg	N02BA01	N02BA	Dr MD 002	Aarau
Patient A	1	Brand X 10 supp 500 mg	N02BA01	N02BA	Dr MD 002	Bern
Patient A	4	Brand X 10 tablets 500 mg	N02BA01	N02BA	Dr MD 001	Aarau
Patient A	1	Brand Y 98 tablets 10 mg	C09AA02	C09AA	Dr MD 001	Aarau
Patient A	2	Brand Y 28 tablets 10 mg	C09AA02	C09AA	Dr MD 001	Aarau
Patient B	3	Brand Y 98 tablets 10 mg	C09AA02	C09AA	Dr MD 007	Vaud
Patient B	4	Brand Z 20 tablets 500 mg	N02BA01	N02BA	Dr MD 008	Vaud

Our analysis did not include any “V” ATC groups (i.e. diagnostic, medical equipment, contrast product, etc.). Moreover, the two following selection criteria were applied at the 4th level ATC:

- more than one specific medicinal product available;
- age difference between the oldest and the newest drug greater than one.

### Variables

The dependent variable was the age of the drug, which we measured as the difference between the date of its inscription on the Swiss Specialities List and 31 December 2006 (analysis time). From an economic point of view, the life cycle of a product begins with its emergence on the market. Thus, the time interval between its registration date and the time of the analysis represents the age of a drug. As explained in the background section, this was our proxy for novelty (i.e. the “younger” the drug, the newer it was considered).

As will be emphasized in the statistical methods subsection, the dataset displayed a complex multilevel structure with five levels, which were both nested and crossed. Explanatory variables were measured at each of these levels.

At the prescription-level, we used two binary variables, one indicating whether the drug was a prescription drug or not, the other taking the value one if the drug was a generic.

At the market-level, we used five variables: number of treated patients (having at least one prescription of a drug belonging to this market), average treatment cost, number of drugs, number of active substances (i.e. the number of ATC 5th level codes), and the Herfindahl-Hirschman index (HHI) [21]. The number of active substances was defined as the number ATC 5th level code available in the market. It has been used as a proxy for the opportunity of real substitution. As for the HHI, it was computed using the market shares of the different brands present in this market.

At the patient-level, we had information related to their age, gender, deductible chosen, and treatment received. From the information on age, we constructed five indicator variables representing five age groups (0-19, 20-39, 40-59, 60-79, 80+). Gender was simply included as a dummy indicating whether the patient was a male and the information on the deductible was also summarized by one indicator variable for whether the patient had chosen a low deductible (lower than CHF 400.-). Since 3rd level ATC categories correspond approximately to different illnesses to treat, we computed a co-morbidity index defined by the number of ATC 3rd level categories applying to each patient.

At the physician-level, we used three indicator variables indicating the physician’s occupation: working in a hospital (but delivering ambulatory care), installed specialist, and installed generalist (internal medicine and generalists). Since data were anonymous, we did not have access to other physicians’ variables (age, rural/urban, etc.).

Finally, at the region-level, we generated nine dummy variables representing each of the nine cantons.

### Statistical methods

The data had a complex multilevel structure, which can be represented as follows [22]:

**Fig. 1:**
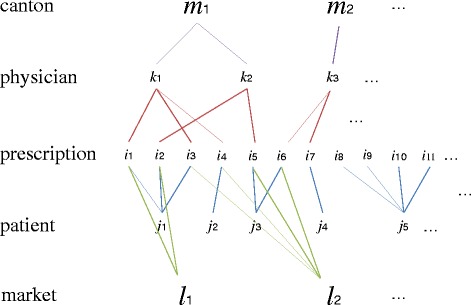
Dataset structure

Figure 1 is self-explanatory and may be simply read as: prescription *i* belonging to drug market *l* and made by physician *k* is addressed to patient *j* in canton *m.*

The dependent variable (i.e. drug’s age) was treated as a continuous variable and was log transformed before analysis as it was extremely skewed. The statistical analyses were carried out using the MLwiN package from within Stata [23, 24]. A non-hierarchical multilevel model with five levels was estimated by Bayesian Markov chain Monte Carlo (MCMC) methods using a burn-in period of 5000 iterations followed by a monitoring period of 10,000 iterations. To ensure a decent pace of convergence, our statistical analyses were conducted on a random – hence representative – subsample of 100,000 insured. Independent improper priors were used for the fixed effects and independent hierarchical Normal for the random effect. Diffuse Gamma hyper-priors were specified for precision. The model may be written in hierarchical notations as follows:

$$ {y}_{ijklm}={\beta}_0+{\beta}_1^{\hbox{'}}{x}_{ijklm}+{u}_j^{id}+{u}_k^{phys}+{u}_l^{market}+{u}_m^{canton}+{\varepsilon}_{ijklm} $$

$$ {u}_j^{id}={\beta}_2^{id\kern0.5em \hbox{'}}{x}_j^{id}+{\delta}_j^{id} $$

$$ {u}_k^{phys}={\beta}_3^{phys\kern0.5em \hbox{'}}{x}_k^{phys}+{\delta}_k^{phys} $$

$$ {u}_l^{market}={\beta}_4^{atc5\kern0.5em \hbox{'}}{x}_l^{market}+{\delta}_l^{market} $$

$$ {u}_m^{canton}={\beta}_5^{canton\kern0.5em \hbox{'}}{x}_m^{canton} $$

$$ {\varepsilon}_{ijklm}\sim N\left(0,{\sigma}_{obs}^2\right) $$

$$ p\left({\beta}_j\right)\propto 1,\kern0.5em j=0,1,\dots, 5 $$

$$ p\left(\left.{\delta}_j^{id}\right|{\sigma}_{id}^2\right)\sim N\left(0,{\sigma}_{id}^2\right) $$

$$ p\left(\left.{\delta}_k^{phys}\right|{\sigma}_{phys}^2\right)\sim N\left(0,{\sigma}_{phys}^2\right) $$

$$ p\left({\delta}_l^{market}\left|{\sigma}_{market}^2\right.\right)\sim N\left(0,{\sigma}_{market}^2\right) $$

$$ p\left(1/{\sigma}_{id}^2\right)\sim Gamma\left(0.001;0.001\right) $$

$$ p\left(1/{\sigma}_{phys}^2\right)\sim Gamma\left(0.001;0.001\right) $$

$$ p\left(1/{\sigma}_{market}^2\right)\sim Gamma\left(0.001;0.001\right) $$

$$ p\left(1/{\sigma}_{obs}^2\right)\sim Gamma\left(0.001;0.001\right) $$

where:

- *x*_*ijklm*_ is a vector of variables measured at the prescription-level (available variables: generic drug (yes/no), Rx (yes/no))
- ${x}_j^{id}$ is a vector of variables measured at the individual-level (available variables: age, gender, deductible ≤400 CH Fr (yes/no), co-morbidity index)
- ${x}_k^{phys}$ is a vector of variables measured at the physician-level (available variables: GP/specialist, hospital/non-hospital)
- ${x}_l^{market}$ is a vector of variables measured at the market-level (available variables: number of patients receiving a drug in this market, average yearly treatment cost, HHI/number of active substances/number of drugs available, cluster means)
- ${x}_m^{canton}$ is a vector of variables measured at the region-level (only dummy variables representing each canton were used)

An unconditional model was estimated to partition the overall variance across the five levels, and the Variance Partition Coefficient (VPC) was computed at each level to assess the proportion of the response variance that lies at each specific level of the model hierarchy [25, 26]. To illustrate the significance of this variance partitioning based on the empirical Bayes estimates of the random effects, we computed at each level of the hierarchy the contrasts between the percentiles P99 and P1, and P75 and P25, in terms of mean drug’s age difference. For this we used a back-transformation to compute the predictions on the original scale [27].

Then, we estimated various conditional models and computed the proportion of explained variation (PEV) at each level to quantify the contribution of patient’s, physician’s, market’s, and prescription’s characteristics to the outcome variance [28]. However, we did not compute the individual variable PEVs as, unless the regressors are all orthogonal, the variable-specific PEVs do not add up to the total level PEV [29]. Instead, to assess the importance of each explanatory variable we computed the contrast (i.e. the difference in outcome values) between two different values of the covariate (e.g. mean drug-age difference between patients in the older and younger age classes, mean drug-age difference between a generic and a non-generic, etc.). Note that the regression coefficients are interpreted as semi-elasticities and, therefore, allow us to compute the percentage change in the outcome value for a unit-change in the regressor value. On the other hand, the back-transformation approach makes it possible to calculate the mean difference of the age of the drugs.

The contrasts were computed by considering all the available explanatory variables at each level, except at the market-level, where only one of the three variables: HHI, number of active substances, and number of drugs was included at a time, as these variables were entangled. Therefore, we estimated three different regressions, in turn, to compute the impact of these three variables. We adjusted for confounding by cluster by including into each regression model the computed cluster means for the variables: age, gender, deductible, co-morbidity index, generic drug, and Rx [30, 31].

Proper convergence of the MCMC algorithm was assessed by inspecting the trace plots, smoothed histograms of the posterior distributions, and auto-correlation functions of the parameters. The goodness of fit of the model was assessed by inspecting histograms of the random effects and residuals, as well as scatter plots of residuals versus predicted mean age of the drug.

All the analyses were carried out using Stata 14.2 (StataCorp LP, 4905 Lakeway Drive, College Station, TX 77845, USA) and MLwiN 2.36 (Centre for Multilevel Modelling, University of Bristol).

## Results

### Variables summary

Table 2 describes the different variables used in this study. Our working subsample contained around 600,000 prescriptions from 328 substitutable markets, prescribed to 100,000 insured, by 9529 physicians in 9 different cantons. Notice that physicians are identified by an anonymous provider number, which may correspond to more than one physician (especially for those working at the hospital).

**Table 2:** Descriptions of the variables used

Variables	Share in % (if not specified differently)	
	Study	Switzerland
Observed prescriptions (599,308 unique values)		
Generic drugs	19.30	n.a.^a^
Rx	78.12	n.a.
Age (mean)	14.59	n.a.
Markets (328 unique values)		
Number of treated patients (mean)	7054	n.a.
Yearly treatment cost (mean)	469	n.a.
HHI (mean)	0.56	n.a.
Number of drugs in the market (mean)	18.5	n.a.
Number of active substances (ATC 5th level) (mean)	2.97	n.a.
Patients (100,000 unique values)		
Age = 0-19	19.6	21.7
Age = 20-39	21.6	27.0
Age = 40-59	28.7	35.1
Age = 60-79	23.5	11.6
Age = 80+	6.7	4.6
Male	43.1	49.0
Deductible >400	34.5	n.a.
Co-morbidity index (ATC 3rd level) (mean)	4.8	n.a.
Physicians (9529 unique values)		
General practitioner (prescriptions’ share in %)	33.30	n.a.
Independent specialist (prescriptions’ share in %)	57.63	n.a.
Hospital (prescriptions’ share in %)	9.07	n.a.
Regions (9 unique values)		
Aarau (patients’ share in %)	9.79	19.33
Basel-Stadt (patients’ share in %)	4.35	6.30
Fribourg (patients’ share in %)	9.29	8.62
Geneva (patients’ share in %)	24.60	14.62
Jura (patients’ share in %)	0.81	2.35
Neuchâtel (patients’ share in %)	6.86	5.72
Ticino (patients’ share in %)	9.47	10.94
Vaud (patients’ share in %)	25.88	22.21
Valais (patients’ share in %)	8.94	9.90

^a^these numbers were not available at the time of our study

Women were over-represented in our sample (around 57%), while the young age categories were under-represented as compared to the Swiss figures. 34% of the individuals represented in our sample chose a deductible higher than 400 CHF and, in average, they consumed drugs from almost 5 different ATC 3rd level codes (proxy for the number of illnesses).

Independent specialists prescribed more than 55% of the delivered drugs, while less than 10% of the prescriptions were written by physicians working in a hospital (drugs prescribed for ambulatory setting, after a hospital discharge or for 1 day surgery, emergency or planed consultations).

The average age of the drugs prescribed was 14.59 years, 19% of them were generics and 78% were Rx. These drugs belonged to 328 distinct substitutable markets, which contained around 7000 insured in average. The average HHI index across these markets was 0.56 while the average number of active substances was almost 3.

Finally, half of the patients came from two cantons: Geneva and Vaud, with an under-representation of the cantons of Aargau, Basel-Stadt and Jura.

### Unconditional variance partitioning and contrasts

Results obtained from the partition of the overall variance across the five levels, as well as the contrasts between the percentiles P99 and P1, and P75 and P25 of the empirical Bayes estimates of the random effects are presented in Table 3. The VPC indicates that most of the response variance lied at the prescription-level and at the market-level (VPC of 42.2% and 50.3% respectively). Also, the contrasts display much higher ranges for these two levels (P99-P1 larger than 50 for both).

**Table 3:** Variance partition coefficients (VPC) and contrasts

Level	VPC (in %)	P99-P1	P75-P25	Max-Min
Prescription	42.3 [39.0; 45.6]	54.3 [51.0; 57.6]	12.4 [11.7; 13.2]	–
Market	50.3 [46.4; 54.2]	52.0 [48.9; 55.1]	16.6 [15.6; 17.6]	–
Patient	4.9 [4.5; 5.3]	13.0 [12.2; 13.7]	2.9 [2.7; 3.1]	–
Physician	2.1 [1.9; 2.3]	11.1 [10.4; 11.8]	1.8 [1.7; 1.9]	–
Region	0.4 [0.3; 0.5]	–	–	4.1 [3.8; 4.3]

Confidence intervals in squared brackets

As for the three remaining levels, patient’s VPC was 4.9, more than twice as large as that of the physician, while region’s VPC was only 0.39%. P99-P1 and inter-quartile range was 13.0 (respectively 2.9) for patient and 11.1 (respectively 1.8) for physician, which also illustrates that a larger amount of output’s variation occurred at the patient level. Finally, note that the contrast between the canton with the highest average drug’s age and the one with the lowest was 4.1 years.

### Conditional variance partitioning

Table 4 displays the results obtained from the estimation of the multilevel regression model with the logarithm of drug’s age as the dependent variable. Since the outcome was measured on the natural logarithmic scale, the slope coefficients multiplied by 100 can be interpreted – ceteris paribus – as the percentage changes in age given a one unit change in the corresponding covariate [32].

**Table 4:** Estimation of the regression coefficients

Variable	Coefficient	Standard deviation	*P*-value	95% credible interval
Prescription-level				
Constant	2.878	0.060	0.000	2.766; 2.989
Rx	−0.656	0.004	0.000	−0.663; −0.649
Generic drug	−0.870	0.003	0.000	−0.876; −0.865
Market-level				
Number of insured	−2.68e-06	1.05e-06	0.002	−4.92e-06; −6.55e-07
Yearly treatment cost	−0.0002	0.00003	0.000	−0.000202; −0.000096
HHI	0.572	0.089	0.000	0.418; 0.753
Number of drugs	−0.0035	0.0008	0.000	−0.005; −0.002
Number of active substances	−0.045	0.012	0.000	−0.068; −0.023
Patient-level				
Age = 0-19	−0.043	0.004	0.000	−0.050; −0.035
Age = 20-39	0.005	0.003	0.045	−0.001; 0.011
Age = 40-59 (reference)	–	–	–	–
Age = 60-79	0.014	0.003	0.000	0.008; 0.019
Age = 80+	0.016	0.004	0.000	0.008; 0.024
Male	−0.006	0.002	0.002	−0.010; −0.002
Deductible >400 CHF	0.009	0.002	0.000	0.004; 0.014
Co-morbidity index	−0.0001	0.0003	0.345	−0.0006; 0.0004
Physician-level				
General Practitioner (ref.)	–	–	–	–
Independent Specialist	0.034	0.004	0.000	0.027; 0.042
Hospital	0.039	0.010	0.000	0.021, 0.057
Region-level				
Aarau (reference)	–	–	–	–
Basel-Stadt	−0.006	0.008	0.251	−0.022; 0.011
Fribourg	0.045	0.008	0.000	0.030; 0.060
Geneva	0.015	0.006	0.010	0.002; 0.027
Jura	0.091	0.016	0.000	0.058; 0.123
Neuchâtel	−0.011	0.009	0.089	−0.028; 0.005
Ticino	0.078	0.008	0.000	0.063; 0.093
Vaud	0.021	0.006	0.000	0.008; 0.033
Valais	0.055	0.008	0.000	0.040; 0.070

Confidence intervals in squared brackets

Based on this interpretation, one can conclude that generics drug’s age was, on average and ceteris paribus, 87% smaller than that of non-generics. Coefficients associated to the Rx variable was negative too, which means that the average age of drugs requiring a physician’s prescription was lower than that of over-the-counter drugs.

Turning to the variables measured at the market level, the only variable displaying a positive coefficient – and, thus, being negatively related to the prescription of novelty – was the Herfindahl-Hirschman index (coefficient of 0.572). Although negative, the coefficients of the four other variables measured at this level were quite small, especially for the number of insured.

In average, older patients took older drugs. Indeed, compared to the reference category (age = 40-59), the coefficients associated to older categories were positive and increasing with age, while the reverse sign was observed for younger individuals (apart from the category 20-39 for which the coefficient is positive but not significant).

Also, males tended to consume more recent drugs than females while individuals who chose a higher deductible took – on average – older drugs.

On average and ceteris paribus, independent generalist prescribed more novelty than independent specialist or physician hired by a hospital. Finally, there was about 10% variation in drug’s mean age between the two most extreme cantons (Neuchâtel and Jura).

The contrasts between low and high values of each covariable on novelty are presented in Table 5 (these contrasts are interpreted as the difference in average drug’s age).

**Table 5:** Contrasts between two different values of the covariate

Variable	Nature of the contrast	Contrast (years)
Prescription level		
Rx	Yes - No	−12.6 [−14.1; −11.1]
Generic drug	Yes - No	−15.2 [−17.0; −13.4]
Market level		
Number of insured	100,000 - 10,000	−5.5 [−6.1; −4.8]
Treatment cost	1000 CHF - 500 CHF	−1.8 [−2.0; −1.6]
Herfindahl-Hirschman index	1 – 0.5	11.6 [10.2; 12.9]
Number of drugs	50 - 1	−6.1 [−6.5; −5.7]
Number of active substances	5 - 1	−6.5 [−7.2; −5.9]
Patient level		
Age	(85+) - (0-19)	0.43 [0.38; 0.48]
Male	Male - Female	−0.16 [−0.18; −0.14]
Deductible	(>400 CHF) - (<=400 CHF)	0.24 [0.21; 0.27]
Co-morbidity index	10 - 1 illnesses	−0.024 [−0.027; −0.021]
Physician level		
Physician’s specialty	Hospital specialist – GP	1.03 [0.91; 1.15]
Region level		
Canton	Jura - Neuchâtel	2.78 [2.46; 3.10]

Confidence intervals in squared brackets

The three largest contrasts were, in increasing order and in absolute value, those resulting from a diminution of the HHI from 1 to 0.5, from the difference between a drug requiring a physician’s prescription and one that did not, as well as from the difference between a generic and a non-generic (11.55, 12.59 and 15.21 respectively).

It is also interesting to note that the contrast obtained from a variation of the number of active substances (5 to 1), the number of drugs (50 to 1), and the number of insured (100,000 to 10,000) in each substitutable market were approximately of the same magnitude (between -5.5 and -6.5).

Finally, the impact of the physician’s speciality was about 1 year, while the contrast between cantons was almost 3 years.

Table 6 shows how much of the total variation was explained by the variables included at the different levels of our multilevel model.

**Table 6:** Variance explained by the variables included at the different level of the model

	Prescription	Market	Patient	Physician
Total VPC (in %)	42.3 [39.0; 45.6]	50.3 [46.4; 54.2]	4.9 [4.5; 5.3]	2.1 [1.9; 2.3]
Residual variance (in %)	34.8 [34.7 34.9]	38.7 [33.2; 45.3]	2.6 [2.6; 2.7]	1.2 [1.2; 1.3]

Confidence intervals in squared brackets

The variables Rx and generic drug captures 5.5% of the total VPC at the prescription-level (42.3-34.8). At the market-level, 11.3% of the total variation was explained by the variables number of insured, treatment costs, and HHI (similar results were obtained for the models using the variables number of drugs or number of active substances instead of HHI). At the patient-level, the covariates age, male, deductible and co-morbidity index explained 2.3% of the total variation, whereas 1.2% percent was captured by the physician’s speciality dummies measured at the physician-level.

## Discussion

Analyzing the drivers of new drugs prescription is a complex task, because several players (doctors, patients, pharmacists, pharmaceutical industries, academic circles, and regulatory authorities) are likely to influence prescribing habits. The scientific literature describing the contributions or drawbacks of specific new substances or formulations is abundant. However, such literature does not address the issue in a holistic way. Apart from the fact that the pharmaceutical industry generally favors innovation for large markets [17] and that patients have only a minor influence since they often lack scientific expertise to make choices [16], very little is known about the determinants of new drugs prescription. To the best of our knowledge, our study is the first addressing all the possible determinants simultaneously.

We found that the main drivers of novelty lied at the market-level and at the prescription-level (more than 92.6% of the variation in drugs’ age occurred at these two levels). As for the remaining 7.4%, 4.9% occurred at the physician-level, 2.1% at the patient-level and 0.4% at the region-level.

More than half of the variation in drugs’ age happened at the market-level and 11.6% was captured by the covariates we included at this level of our multilevel model. Results indicate that more recent drugs are prescribed in markets that are costlier, less concentrated, include more insured, provide more drugs and include more active substances. For instance, a market containing 100,000 insured prescribed, on average and ceteris paribus, drugs that were 5.5 years younger than those prescribed in a market containing 10,000 insured. The variable representing market concentration (i.e. HHI) had a strong effect: drugs prescribed were on average 11.5 years older in markets with a HHI of 1.0 compared to those with a HHI of 0.5. These observations suggest that producers tend to develop new products if there is a sufficient target turn-over and concurrency. The residual variance of 38.72% that was not due to the covariates we included was likely related to the age of the pioneers (i.e. first drug introduced in the market).

The prescription-level explained another great part of the variance (42.2%), with more novelty in drugs requiring physicians’ prescription and generics. Over-the-counter drugs were about 12.5 older, probably because they correspond to reformulations of well-known substances (pioneer drugs). As for generic drugs, they were registered much earlier than non-generics (15.2 years in average). This latter effect was not surprising since generic drugs can only enter the market after patents’ expiration. The important thing here is that the effect of all studied variables was adjusted for this phenomenon.

At the patient-level, elderly and females tended to consume older drugs, but with a low contrast (i.e. less than 6 months) between the two extremes categories (i.e. 0-19 and 85+). Our results confirm that most patients suffered from multiple morbid conditions, especially among old people [33]. However, we found that the number of morbid conditions, as measured by our co-morbidity index, had little impact on the age of prescribed drugs, after partialling out the effect of all other variables*.* Deductible did not have a strong impact on receiving new drugs either. As patients have to pay a quote-part of only 10%, this did not impact their choice of consuming newer, and maybe more expensive, drugs. Therefore, it seems that cost of treatment is not a risk to create inequities between patients in the Swiss model of funding.

At the physician-level, generalist tended to prescribe newly registered drugs more frequently than hospital or independent specialists did. Although relatively weak (1 year difference), this contrast appears counter-intuitive. One explanation could be that specialists prescribe drugs in clinical and therapeutic areas where they have expertise and that these areas have observed fewer market entries in the early 20’s compared to other areas [6, 34]. Unfortunately, we did not have enough information about physicians to check this hypothesis. This contrast can also be related to the prescription of reformulated drugs by general practitioners (which were considered as novelty in this study), more convenient for chronic administration or for patients with cognitive decline, and increase patients’ adherence [35].

Finally, at the region-level the contrast in terms of novelty between two extreme cantons was relatively high (i.e. almost three-years difference between the Neuchâtel and Jura). This suggests that regional opinions might be influenced. Cantonal differences in drugs prescription was also observed by other authors [36]. We did not found the amounts spent into marketing efforts to understand if there were correlated to these regional differences. However, we noted that Catholic cantons (Ticino, Valais, Fribourg, Jura), which are typically considered as more traditional, consumed older drugs. This raises the question of fair geographic access to new products.

Our study has some limitations, which might be fulfilled by further researches. First, we did not have access to detailed information about physicians and, thus, were not able to test the impact of “contagion through social networks in new drug uptake” for instance [6]. Moreover, our study was cross-sectional whereas such contagion assumption would require a longitudinal survey to analyze the geographic dynamic of physicians’ prescriptions habits. We adopted a complex multilevel structure and adjusted our results to avoid confounding by clusters but decided not to use an over-sophisticated model.

Further researches would be required to deepen the analysis of the interactions between drugs’ markets and physicians [37], which might show mixed effects at physicians’ level (e.g. simultaneous preferences for some old medicines and for other recent). It would also be interesting to analyze if the drivers of new drugs prescription are homogeneous or different among types of innovations in each drugs’ markets (new active substances, new formulations, generics). Further studies could also address the question of the equity to access to specific innovations. Finally, it would be interesting to repeat the analyses within one defined disease area and range of drugs commonly used (e.g. cardiovascular disease) and compare the results with those obtained in the present study.

## Conclusion

Producers have two main ways to introduce new drugs: diversification of dosage and repackaging of already existing substances or development of new similar substances. In Switzerland, there are several hundred new products introduced each year on the healthcare market. It thus seems important to understand the mechanism leading to the prescription of these new drugs.

This study provided a global picture regarding the drivers of new drugs prescription. Our results indicated that regulation of the demand has low impact, with little variation explained at the patient-level and physician-level. In contrary, the market structure (e.g. end of patent with generic drugs apparition, concurrence among producers) had a strong contribution to the variation of prescribed drugs age.

## Abbreviations

ATC: Anatomical therapeutic chemical
HHI: Herfindahl-Hirschman Index
MCMC: Markov Chain Monte Carlo
PEV: Proportion of explained variation
Rx: Prescription drug
VPV: Variance partition coefficient
